# Coronary arterial fistulas

**DOI:** 10.1186/1750-1172-1-51

**Published:** 2006-12-21

**Authors:** Shakeel A Qureshi

**Affiliations:** 1 Evelina Children's Hospital, Guy's & St Thomas's Hospital Foundation Trust, London, UK

## Abstract

**Abstract:**

A coronary arterial fistula is a connection between one or more of the coronary arteries and a cardiac chamber or great vessel. This is a rare defect and usually occurs in isolation. Its exact incidence is unknown. The majority of these fistulas are congenital in origin although they may occasionally be detected after cardiac surgery. They do not usually cause symptoms or complications in the first two decades, especially when small. After this age, the frequency of both symptoms and complications increases. Complications include 'steal' from the adjacent myocardium, thrombosis and embolism, cardiac failure, atrial fibrillation, rupture, endocarditis/endarteritis and arrhythmias. Thrombosis within the fistula is rare but may cause acute myocardial infarction, paroxysmal atrial fibrillation and ventricular arrhythmias. Spontaneous rupture of the aneurysmal fistula causing haemopericardium has also been reported. The main differential diagnosis is patent arterial duct, although other congenital arteriovenous shunts need to be excluded. Whilst two-dimensional echocardiography helps to differentiate between the different shunts, coronary angiography is the main diagnostic tool for the delineation of the anatomy. Surgery was the traditional method of treatment but nowadays catheter closure is recommended using a variety of closure devices, such as coils, or other devices. With the catheter technique, the results are excellent with infrequent complications.

**Disease name and synonyms:**

Coronary arterial fistulas

Coronary arterial fistulas or malformations

## Background

### Definition

A coronary arterial fistula (also known as coronary arteriovenous malformation) is a connection between one or more of the coronary arteries and a cardiac chamber or great vessel, having bypassed the myocardial capillary bed.

### Epidemiology

This is a rare abnormality and usually occurs in isolation [1]. Its exact incidence is unknown. The majority of the fistulas have a congenital origin, but may occasionally be detected after cardiac surgery, such as valve replacement, coronary artery bypass grafting and after repeated myocardial biopsies in cardiac transplantation [2,3].

### Morphology

**Figure 1:**
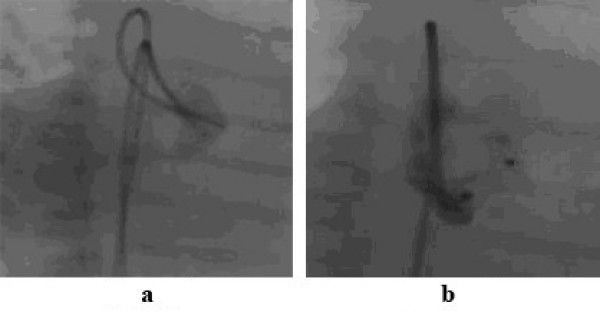
1a shows a dilated fistula between the proximal right coronary artery and right atrium. Such a fistula is suitable for an occlusion device such as the Amplatzer duct occluder (Figure 1b).

**Figure 2:**
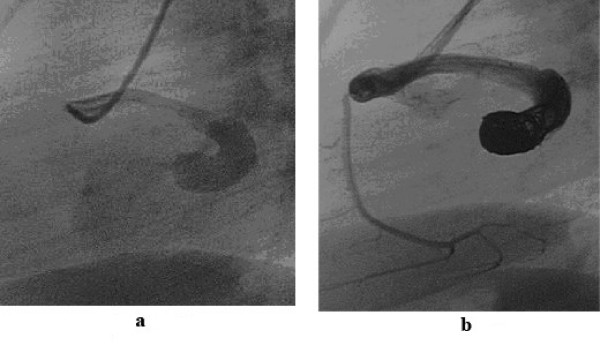
2a shows a fistula between the proximal part of the right coronary artery and drains to the right atrium. There is a stenosis at the entry point into the right atrium. Such a fistula may be suitable for closure with coils or duct occluder type of device. In Figure 2b, the fistula has been closed with coils.

**Figure 3:**
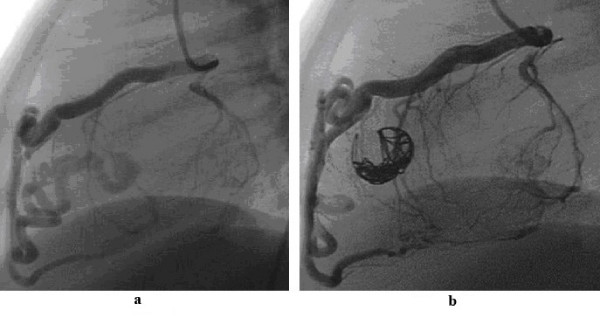
3a shows a very tortuous fistula between the left anterior descending coronary artery and the right ventricle. Such a fistula is suitable for controlled-release coils (3b) rather than devices.

**Figure 4:**
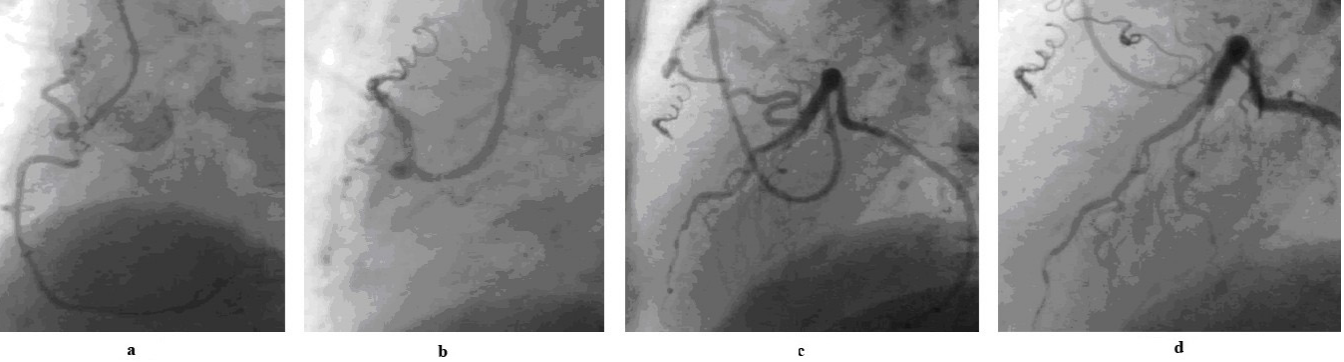
4a shows a tortuous small fistula between the proximal right coronary artery and the pulmonary trunk, which has been closed with controlled-release coils (4b). Figure 4c shows a tortuous fistula between the left anterior descending coronary artery and the pulmonary trunk in the same patient, which has been closed also with controlled-release coils (4d).

The feeding artery of the fistula may drain from a main coronary artery or one of its branches and is usually a dilated and tortuous artery terminating in one of the cardiac chambers or a vessel. The more proximal the feeding artery originates from the main coronary artery, the more dilated it tends to be. If the fistula drains to the right atrium with a proximally arising feeding artery, it tends to be considerably dilated but less tortuous (Figure 1a, b and Figure 2a, b). If there is a more distal origin of the feeding artery, and in particular when the fistulas originate from the left coronary artery and drain to the left ventricle, they may be very tortuous, presenting a challenge for catheter closure (Figure 3a, b). However, in the less frequently encountered right coronary artery to coronary sinus drainage, the fistula vessel may be large and very tortuous. It is important to note that there may be multiple feeding arteries to a single coronary arterial fistula drainage point or there may be multiple drainage sites [2]. The fistulas originate from the right coronary artery in about 52% of cases, the left anterior descending coronary artery being the next most frequently involved in approximately 30% of cases and the circumflex coronary artery in about 18% of cases [4]. Over 90% of the fistulas from either coronary artery drain to the right side of the heart and the remainder drain to the left side of the heart [5]. In the right heart, drainage occurs most frequently to the right ventricle (in about 40%), followed by the right atrium, coronary sinus, and pulmonary trunk. Multiple fistulas between the three major coronary arteries and the left ventricle have also been reported [6]. In adults, occasionally fistulas may be encountered which originate from both the coronary arteries and drain into the pulmonary trunk. These fistulas may cause angina and require closure (Figure 4a, b).

### Pathophysiology

When the fistula drains to the right side of the heart, the volume load is increased to the right heart as well as to the pulmonary vascular bed, the left atrium and the left ventricle. When the fistula drains into the left atrium or the left ventricle, there is volume overloading of these chambers but no increase in the pulmonary blood flow. This results in different echocardiographic appearances of dilation of different cardiac chambers due to the shunts. The size of the shunt is determined by the size of the fistula and the pressure difference between the coronary artery and the chamber into which the fistula drains. Occasionally congestive cardiac failure occurs and very rarely, in adults, myocardial ischaemia may occur.

## Clinical features

Coronary arterial fistulas are usually asymptomatic in the first two decades, especially when they are haemodynamically small. Indeed, a small number may close spontaneously. After this, the frequency of both symptoms and complications increases [7]. Complications include 'steal' from the adjacent myocardium causing myocardial ischaemia, thrombosis and embolism, cardiac failure, atrial fibrillation, rupture, endocarditis/endarteritis and arrhythmias [1,4,8,9]. Thrombosis within the fistula is rare but may cause acute myocardial infarction, and atrial and ventricular arrhythmias [10]. Spontaneous rupture of the aneurysmal fistula causing haemopericardium has also been reported [11].

The fistulas may increase in size over time, although this does not occur invariably. They may form a short and direct connection with a chamber or a large vessel, or form complex long tortuous and aneurysmal cavities. The largest shunts occur when the coronary artery connects to the right side of the heart rather than the left heart chambers.

### Symptoms and signs

The majority of the patients are asymptomatic. In the older patients, symptoms may include dyspnoea or angina of effort and occasionally arrhythmias. Patients with large left-to-right shunts may develop congestive cardiac failure, especially in infancy and occasionally in the neonatal period [12]. If angina is reported, it may be due to coronary artery steal [13].

Most patients are referred because of an asymptomatic continuous murmur, loudest over the praecordium, which may be thought to be due to patent arterial duct. However, the murmur is heard over the mid-chest, or even lower, rather than below the left clavicle and typically peaks in mid to late diastole rather than systole. If the fistula connects to the left ventricle, only an early diastolic murmur may be heard. Some patients with large shunts may present with signs of congestive cardiac failure and angina, usually at the two extremes of life.

### Differential diagnosis

The main differential diagnosis is patent arterial duct, although other arteriovenous shunts need to be excluded.

## Diagnostic methods

The electrocardiogram and chest x-ray are unhelpful usually, although the electrocardiogram may show the effects of left ventricular volume overload and occasionally ischaemic changes. When the electrocardiogram is normal and if the patient is old enough to exercise on the treadmill with electrocardiographic monitoring, ischaemic ST-segment changes may become apparent [13]. Generally the chest x-ray is normal, but occasionally moderate cardiomegaly may be present when there is a large left-to-right shunt.

Two-dimensional and colour Doppler echocardiography are helpful in demonstrating dilation of the affected coronary artery and on colour flow mapping may show the site of drainage, but it is difficult to define the detailed anatomy of the fistula with this technique. Clues may be present when the feeding coronary artery is enlarged or ecstatic or tortuous. On colour Doppler flow imaging, large flow may be seen at the origin or even along the length of the vessel and indeed flow into the right heart chambers may also be visualised.

Magnetic resonance imaging may also help in confirming the diagnosis, as the proximal coronary arteries or even the whole length of the fistula vessel may be seen. In older patients, stress thallium studies may be used to assess myocardial ischaemia before and after treatment.

Procedural options can be optimised by careful identification of the number of fistulous connections, nature of the feeding vessel or vessels, sites of drainage, and quantification of myocardium at risk for injury or loss. The goal of treatment is the occlusion of the fistula, whilst preserving normal coronary blood flow.

The main diagnostic technique is cardiac catheterisation and angiography. Initial diagnostic catheterisation is needed to assess the haemodynamic significance of the fistula and to provide detailed anatomy of the fistula, in particular, the size, the origin, the course, presence of any stenoses and the drainage site. This helps to plan the appropriate treatment. A preliminary aortic root angiogram in a 'laid-back view' helps in determining which coronary artery to catheterise selectively [14]. Selective coronary angiography of both the coronary arteries is needed to confirm the diagnosis, the detailed anatomy and the presence of multiple fistulas. However, this should only be performed when definitive treatment such as an interventional procedure or surgery is planned. The angiographic views include right anterior oblique, anteroposterior, left anterior oblique, left anterior oblique with caudo-cranial angulation and left lateral projections.

## Management

The indications for treatment of coronary arterial fistulas include the presence of a large or increasing left-to-right shunt, left ventricular volume overload, myocardial ischaemia, left ventricular dysfunction, congestive cardiac failure and for prevention of endocarditis/endarteritis.

The treatment options for coronary arterial fistulas include surgery or catheter closure. Surgery involves internal closure of the fistula within the receiving chamber or vessel whenever feasible, but when the fistula is associated with a large aneurysm of the feeding artery, it may need to be ligated from within the aneurysm. Surgery is not risk free: it is associated with a low morbidity and mortality rate ranging from 0 to 6% [15,16]; myocardial infarction may occur in less than 5% of cases and there is a risk of recurrence of the fistula [17]. The reason for the recurrence includes the fact that there may be multiple fistulas present which are difficult to deal with by surgery. Complete occlusion of the fistula may be achieved in >95% of cases after surgery although the exact incidence of residual fistulas is not known.

Catheter closure of the fistulas is now considered to be an effective and safe alternative to surgery [2,18,19]. The aim of catheter closure is to occlude the fistula artery as distally and as close to its termination point as possible, so as to avoid any possibility of occluding branches to the normal myocardium. If, however, embolisation is effected too distally and if there is no significant stenosis within the vessel, the embolisation device could migrate beyond the fistula into the pulmonary circulation. Whichever technique of catheter closure is used, the occlusion should be at a precise point. Different types of embolisation materials can be used such as detachable balloons, stainless steel coils or platinum microcoils [2,19-23]. The technique may be influenced by several factors such as the age of the patient, the morphology of the feeding arteries and the location of the fistula. Many types of equipment should be available in the catheterisation laboratory for closing the fistulas. These include a selection of non-tapered catheters, balloon catheters, Tracker or Ferret catheters, different types of floppy or superfloppy coronary guidewires of 0.014" calibre, different types and sizes of coils (conventional Gianturco coils and controlled-release coils), detachable balloons and a variety of occlusion devices, normally used to close atrial or ventricular septal defects or patent arterial ducts or collateral vessels.

The choice of the equipment and the technique depends on the morphology of the fisulas. The factors include their tortuosity, the presence of high flow in the fistula, aneurysmal dilation of the feeding vessel and the point of intended occlusion. Other important determinants include the age and size of the patient, the catheter size that can be used in the patient, the size of the vessel to be occluded and the tortuosity of the catheter course to reach the intended point of occlusion. For example if the route to the target vessel is tortuous, then superfloppy guidewires combined with 3 Fr Tracker or Ferret catheters are recommended. If there is a high flow through the fistula, then stop-flow technique with a balloon will need to be used during deployment of a coil or a device. Because of the need for precise occlusion, a potentially reversible technique is preferable.

### Technique of catheter closure

Access is usually needed in both the femoral arteries and one femoral vein and 5 Fr sheaths are inserted initially. The reason for using both the femoral arteries is that check angiograms can be performed through a catheter from one femoral artery, whilst a device is being implanted from the other. Also in a high flow fistula, a balloon catheter can be inflated through the second arterial access to control deployment of an occluding device.

Judkins left and right coronary catheters are used for selective angiography for defining the anatomy. A Berman or Swan-Ganz type of balloon catheter is passed and the balloon inflated with contrast or carbon dioxide to temporarily occlude the vessel. The purpose of this is to test for ischaemia after inflation of the balloon for 5–10 minutes. In the absence of ischaemic changes, the site where the balloon has been kept inflated or beyond are acceptable sites for occlusion of the fistula.

In the absence of any ischaemic changes, a guiding coronary catheter is positioned in the artery. With the help of a 0.035" standard guidewire advanced into the fistula, the guiding catheter may be passed to the point of intended occlusion in cases with a relatively straight course of the feeding artery. In this case, either Gianturco or Cook-PDA coils can be deployed through this catheter to achieve occlusion. Gianturco coils of 0.038" calibre require non-tapered catheters of 4 Fr or 5 Fr size for their delivery. The coil should be up to 30% larger than the vessel to be occluded at the point of occlusion to avoid inadvertent embolisation of the coil. Once the first coil is in correct position, different sizes of coils can be deployed subsequently to form a tight nest.

Positioning such stiff guiding catheters in a distal location in a tortuous fistula may be difficult and hazardous. In these patients with multiple bends in the feeding artery, it may be preferable to use controlled-release platinum micro-coils of 0.018" calibre, which can be deployed through a co-axial 3 Fr Tracker or Ferret catheter passed through the guiding catheter. Such catheters can be manipulated through tortuous arteries into very distal locations over superfloppy, steerable 0.014" coronary guidewires, and through them interlocking-detachable coils (IDC) or detachable coil system (DCS) coils can be deployed. Controlled-release coils make the procedure more controlled and potentially reversible. With a high flow fistula, temporary balloon occlusion may be needed during the deployment of the coils [24,25]. Multiple coils can then be deployed serially through these catheters to form a tight nest before deflating the balloon [24]. Controlled release coils can be positioned and withdrawn back into the catheter, if the final position is not satisfactory. The coils are not fibred and so thrombosis takes a considerably longer time than with fibred coils.

In some patients, it may be easier to enter the fistula easily from the right side of the heart. These may be suitable for occlusion with an Amplatzer occluder type of devices, such as a vascular plug, a duct occluder or an atrial or ventricular septal occluder [12,26-29]. The fistula vessel should be large, have easy and straight access from the right heart, if needed with the help of an arteriovenous guidewire circuit, and allow a guiding sheath to be passed into the vessel for the occlusion device. In these, either femoral venous or internal jugular venous access is used.

Detachable balloons are rarely used nowadays. They can be floated out with the arterial flow and achieve immediate occlusion and then the balloon is detached [20,23]. They are complex to use and require large introducer catheters (6–8 Fr). Early deflation and premature detachment of these balloons are further problems, which have made most operators avoid using them.

After occluding the main fistulous vessel, repeat selective coronary angiography in both the coronary arteries is essential as a second branch feeding the fistula or multiple feeding vessels may be visualised, which may also need occlusion at the same procedure (figure 4c &4d).

With catheter closure techniques, complete occlusion of the fistula may be achieved in >95% of the patients. In the remaining patients, either further procedures may be required to close the fistulas or they may be managed conservatively if the residual fistulas are small. The main complications include either premature deflation of a detachable balloon, inadvertent coil embolisation, transient T-wave changes, transient bundle branch block and myocardial infarction. All the complications are rare, apart from inadvertent coil migration, which may occur as a result of high flow in the large fistulas or with undersized coils [24]. Even if the coils do migrate, they can be retrieved with snares.

## Discussion

The majority of coronary arterial fistulas are asymptomatic in the early years. Larger fistulas may result in congestive cardiac failure or angina at the extremes of life, in infants or middle aged or older adults. Occasionally these fistulas have been detected prenatally, in which case they may cause congestive cardiac failure soon after birth [30]. If fistulas are detected in infancy and are asymptomatic, conservative management is appropriate, as very rarely, spontaneous closure of a small fistula has been reported [31]. If the fistula does not close with growth of the patient, then the older the patient, the easier the catheter procedure becomes technically. These fistulas are infrequently encountered, so most operators will only deal with a small number of cases of coronary arterial fistulas each year. The aim of the catheter procedure should be to achieve complete occlusion at as distal a location as possible in the fistulous vessel. Specialised techniques and equipment are needed and occasionally a combination of techniques is required. The availability of a wide range of equipment makes it possible to occlude a majority of the fistulas. The main complication is inadvertent embolisation of the devices, but even then it is possible to retrieve the devices with goose-neck snares.

## Conclusion

It is important to use a technique suitable for the size and the location of the coronary arterial fistula and the size of the patient. A wide range of equipment should be available to deal with all the fistulas, as well as possible complications of the techniques. Excellent results can be achieved by the catheter closure techniques. The technique of catheter closure allows further arterial feeding vessels to be discovered by selective coronary angiography at the end of the procedure and if such multiple feeding vessels are noted, these can also be occluded.
